# Graphical User Interfaces for Molecular Dynamics—Quo Vadis?

**Published:** 2009-09-23

**Authors:** B. Knapp, W. Schreiner

**Affiliations:** Unit for Medical Statistics and Informatics - Section for Biomedical Computer Simulation and Bioinformatics Medical University of Vienna. Email: bernhard.knapp@meduniwien.ac.at

**Keywords:** molecular dynamics, graphical user interface

## Abstract

In the past years an increasing number of graphical user interfaces for Molecular Dynamics (MD) were presented and concomitantly, more and more Molecular Dynamics studies were published. With the easier application of MD software packages the field runs the risk however, of being pervaded with unreliable results. Therefore, possible benefits and caveats have to be carefully balanced. Here we outline in which respects a broader access of MD via graphical user interfaces may help to increase the usability of Molecular Dynamics simulations while maintaining their quality.

Molecular Dynamics^1^ (MD) is a computational technique which solves Newton’s equations of motion to predict the movements of atoms within molecules over time. This technique can give insights into spatial arrangement of systems where experimental approaches fail due to limited resolution (electron tomography), demanding and unfeasible crystallisation (x-ray crystallography), or limited size (NMR). Furthermore, MD provides the spatial dynamics of a system over time, giving new insights and appreciations of molecular functions relevant to biological processes such as, e.g. T cell activation.^2^,^3^

To supply MD to a broader audience, graphical user interfaces (GUIs) are developed. Such GUIs exist for CHARMM,^4^ e.g. the web-based CHARMM-GUI^5^ providing a membrane builder, or also CHARMMing,^6^ including additional programs to define structures not supported by standard CHARMM and a job manager for grid computing. For the AMBER^7^ MD package the software Glycam Biomolecule Builder^8^ is available, allowing to build new biomolecules and directly submit them to AMBER. For the MD Software NAMD^9^ the NAMD-GUI^10^ is provided as plug-in for the 3D viewer VMD.^11^ Also for the GROMACS^12^ MD package different GUIs exist: For example, jSimMacs^13^ is a Java implementation and provides an interactive 3D representation and remote access capability for MD simulations. GROMITA^14^ is a cross-platform perl/tcl-tk based solution, GUIMACS^15^ is Java based and GROMACS-GUI^16^ is developed in C++. For a detailed comparison between GROMACS GUIs see Roopra et al.^13^

Using one of them, scientists even without technical background and Linux command line knowledge are able to perform MD and thereby support their research with additional data on structures and functions. However, this leads to a fundamental issue: With MD methods becoming more and more easy to handle, fast and freely accessible, a growing audience of researchers will come in contact with the field, as indicated by the increasing number of publications in MD (see Fig. 1). Some of those simulations may be carried out by “trial and error”, clicking on buttons until the simulation starts somehow. Such lack of sound knowledge about what is really calculated in background may lead to incorrect settings and/or results: insufficient time for energy minimisation and position restraining, too short cut offs, water shells with inappropriate sizes et cetera. These and many more parameters need to be carefully chosen for each system.^17^ Otherwise, unreliable and irreproducible simulations may result, which run the risk of endangering the scientific reputation of the field.

One could argue that the peer review system should be able to spot and filter out such publications. However, this may be wishful thinking since most reviewers will concentrate on the manuscript only and not examine the original data or background information for reasons of time. For experts in MD, weaknesses in the description of methods etc. would still be noticeable and they might ensure clarification. However, as MD becomes “just a tool”, all reviewers of a paper (using MD among other methods) might have a “biomedical” focus and lack personal expertise in MD. In such a case, results might pass review as long as they look plausible and fit into the picture, even if serious questions regarding reliability would be appropriately tackled. Hence, there are opinions in the community which entirely decline the development of any kind of graphical user interface, following the ulterior reasoning that the additional barrier of command line handling may keep off inexperienced users from MD techniques. By this way only researchers willing to invest time and effort in learning the ropes of MD will be able to carry out simulations.

It is obvious that researchers able to perform MD properly from a command line level may also use GUIs: they know “what is going on behind the scene” and may still benefit from easier handling (select instead of type, no miss-typing, automatic naming of serial files etc. (if it is a really potent GUI). Users approaching solely via the GUI at any rate run a higher risk. From the authors’ point of view, in order to curb that risk, four major perspectives should be discussed by the community, as itemized in the following (and formulated as guidelines in Box 1 and Box 2):

1. For all kinds of simulations standardized and exchangeable parameter files (e.g. in XML-format) should be provided with each submission. Supplying such files it will be visible at a glance what was done in the simulation and disguising of grubby settings will not be possible. For more complex situations even virtual machines containing all necessary environment parameters could be provided. GUIs could output such files. Similar ideas, however for much more general purposes, were already put forward.^18^
2. Expert systems could be implemented in MD-GUIs to guide the inexperienced user and help to avoid wrong decisions. Similar thoughts were suggested by an anonymous reviewer to one of our manuscripts. These systems could even learn from past parameter settings which have led to exploding, crashing or hang up simulation runs. However, it seems obvious that such systems can never fully replace a cautious and reflective human.
3. Repetitive MD simulations of the same system (using slightly different initial forces) can help to sample an extended part of the solution space (up to thousands of ns in total), avoid random errors and yield results more reliable due to more sufficient sampling.
4. Whenever MD is applied to a molecular system where it has not yet reached the phase of mature and routinely performed work but is still investigative in character, MD results published should be supported and/or matched with experimental data. This will help reviewers to decide whether a simulation is reliable or not. In this context experimental data does not necessarily mean x-ray crystallography or NMR. Also binding studies or observed immunological processes may support MD results.^2^,^3^ However, if no experimental data is available, it may also be an opportunity to employ other computational methods such as normal mode analysis (NMA) to validate MD results.^19^

Along these lines molecular dynamics—also if run via GUIs—will come of age and find its place as standard technique in wet laboratories. There it will complement and—may be in future—replace parts of the experiments.

## Figures and Tables

**Figure 1.**
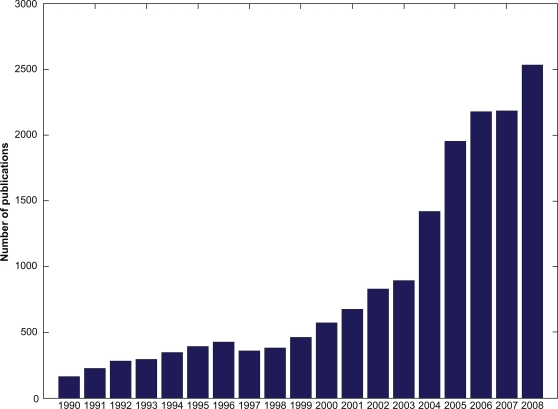
Number of published MD papers per year. Data based on Pubmed search in July 2009.

**Box 1.**
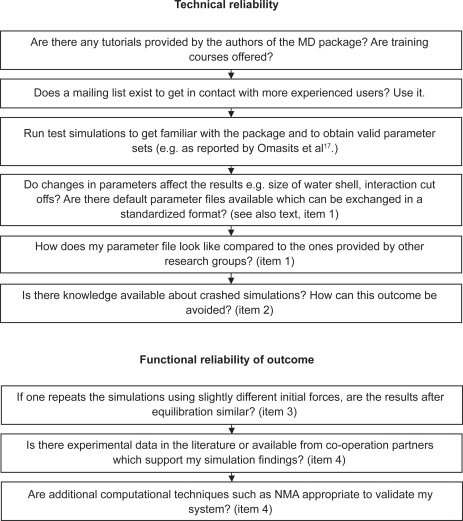
Guidelines and questions part 1: How inexperienced users can assure reliable simulations, when not using GUIs.

**Box 2.**
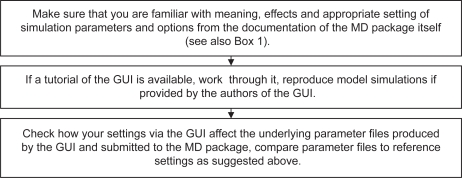
Guidelines and questions part 2: How inexperienced users can assure reliable simulations, when using GUIs.
